# Catecholamine signaling that modulates cerebellar operations in cognition

**DOI:** 10.1038/s41386-020-00854-x

**Published:** 2020-09-09

**Authors:** Hirofumi Fujita, Erik S. Carlson

**Affiliations:** 1 Department of Otolaryngology–Head and Neck Surgery, Johns Hopkins University, Baltimore, MD USA; 2 Department of Psychiatry and Behavioral Sciences, University of Washington, Seattle, WA USA; 3 Geriatric Research, Education and Clinical Center, Veteran’s Affairs Medical Center, Puget Sound, Seattle, WA USA

**Keywords:** Working memory, Classical conditioning, Neurotransmitters

Catecholamine signaling characterizes the mesocorticolimbic system underlying affective, cognitive, and executive functions, and is targeted by pharmacological therapies for patients with neuropsychiatric disorders. Recent studies highlight the importance of catecholaminergic signaling in cerebellum for pathophysiology and therapeutics of cognitive symptoms.

The cerebellum is implicated in a broad range of neuropsychiatric disorders including autism, schizophrenia, mood disorders, and Alzheimer’s disease [1]. Anatomically, these observations are supported by extensive and closed connections between the cerebellum and forebrain regions including prefrontal cortex, basal ganglia, and hippocampus [1, 2]. Changes in cerebello–forebrain connectivity have been characterized in patients with psychiatric illnesses, and cerebellar stimulation has beneficial therapeutic effects for cognitive and affective symptom domains in both animal models and human patients with autism and schizophrenia [1, 3].

While catecholamines are typically thought to act primarily in forebrain regions, they are also abundant in cerebellum. Cerebellum consists of uniform circuit motifs (Fig. 1), and thus basic circuit mechanisms elucidated in classical studies of motor learning can be applied to cognitive cerebellar outputs. Catecholamines are present in at least three different sites in these circuit motifs (Fig. 1). First, catecholamines provided by extracerebellar sources (e.g., locus coeruleus) act on climbing fiber–Purkinje cell (PC) synapses, which modulate cerebellar cortical plasticity. Second, PC dendrites release dopamine in an autocrine/paracrine fashion, and PCs change excitability through their dopamine receptors in vitro [4]. Catecholamine-modulated PC activity directly affects glutamatergic output from cerebellar nuclei (CN), via their powerful inhibitory PC–CN synapses. Third, catecholamines act on dopamine and norepinephrine receptors of CN neurons. These induce metabotropic signaling, resulting in altered excitability of glutamatergic output neurons, glycinergic local neurons, and GABAergic preolivary neurons of CN, and ultimately alter cognitive cerebellar output [5].

**Fig. 1:**
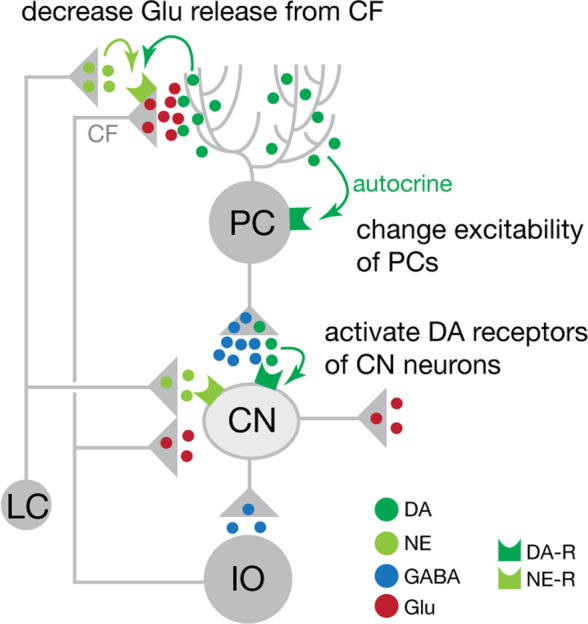
Sites of specific catecholamine actions in cerebellar circuits. See text for explanation. CF climbing fiber, CN cerebellar nucleus, DA dopamine, IO inferior olive, LC locus coeruleus, NE norepinephrine, PC Purkinje cell, -R receptor.

Modern circuit dissection approaches have been used to investigate in vivo roles for CN neurons expressing dopamine receptors and tyrosine hydroxylase (TH) in PCs in cognition [5, 6]. CN neurons that express dopamine D1 receptor (D1R) are largely inhibitory and are concentrated in a cognitive region of the CN, the dentate nucleus, in both mice and humans [5]. Selective chemogenetic manipulations of D1R+ neurons demonstrated their necessary involvement in spatial navigation memory, response inhibition, social recognition memory, working memory, and prepulse inhibition in mice [5]. TH+ PCs are also distributed at cognitive parts of the cerebellar cortex, the posterior vermis, and hemispheres [6]. We produced a transgenic mouse model for selective knockout of *Th* from PCs, disabling PC catecholamine synthesis and thus their autocrine/paracrine and possibly, terminal release of dopamine [4, 6]. Mutant animals had impairments in behavioral flexibility, response inhibition, social recognition memory, and associative fear learning [6]. In either manipulation, no gross sensorimotor deficits were observed [5, 6]. These examples indicate that catecholaminergic signaling is necessary for cerebellar cognitive functions. Importantly, these cerebellum specific manipulations underscore significance of cerebellar catecholamine-related symptoms and reveal a novel locus for pharmacological intervention.

More experiments are needed to elucidate catecholamine signaling in cerebellar circuits, and provide a better, mechanistic understanding of their role in pathophysiology and treatment of cognitive and affective deficits in neuropsychiatric disorders.

## Funding and disclosures

This work was supported by Department of Veteran’s Affairs (salary for ESC) and the National Institutes of Health Grant No. R01-MH116883 to ESC. HF is supported by NIH grant NS095232 and NS105039 awarded to Dr. Sascha du Lac. The authors declare that the submitted work was carried out without the presence of any personal, professional, or financial relationships that could potentially be construed as a conflict of interest.
